# Space and time in the child's mind: metaphoric or ATOMic?

**DOI:** 10.3389/fpsyg.2013.00803

**Published:** 2013-11-05

**Authors:** Roberto Bottini, Daniel Casasanto

**Affiliations:** ^1^Department of Psychology, University of Milan-Bicocca, Milan, Italy; ^2^Department of Psychology, University of Chicago, Chicago, IL, USA

**Keywords:** conceptual metaphor, ATOM, space, time, children

## Abstract

Space and time are intimately linked in the human mind, but different theories make different predictions about the nature of this relationship. Metaphor Theory (MT) predicts an asymmetric relationship between space and time. By contrast, A Theory of Magnitude (ATOM) does not predict any cross-dimensional asymmetry, since according to ATOM spatial and temporal extents are represented by a common neural metric for analog magnitude. To date, experiments designed to contrast these theories support MT over ATOM, in adults and children. Yet, proponents of ATOM have questioned whether some of the observed cross-dimensional asymmetries could be task-related artifacts. Here we conducted a test of the asymmetric relationship between space and time in children's minds, equating the perceptual availability of spatial and temporal information in the stimuli more stringently than in previous experiments in children. Results showed the space-time asymmetry predicted by MT. For the same stimuli (i.e., snails racing along parallel paths), spatial information influenced temporal judgments more than temporal information influenced spatial judgments. These results corroborate previous findings in Greek children and extend them to children who speak Dutch and Brazilian Portuguese. The space-time asymmetry in children's judgments is not due to task-related differences in the perceptual availability of spatial and temporal information in the stimuli; rather, it appears to be a consequence of how spatial and temporal representations are associated in the child's mind.

## Introduction

Space and time are intimately linked in the human mind. This relationship, which has long been a topic of philosophical inquiry (Locke, 1689/1995; Bergson, 1889/1910), has also been the subject of psychological experiments since the nineteenth century (e.g., Mach, 1886/1897; Benussi, 1913; Piaget, 1927/1969; Boroditsky, 2000; Núñez and Sweetser, 2006; Vicario et al., 2007; Casasanto and Boroditsky, 2008; Miles et al., 2010; Srinivasan and Carey, 2010; Santiago et al., 2011; Ulrich et al., 2012).

In the twenty-first century, two theories have motivated numerous experiments on relationships between space and time: A Theory of Magnitude (ATOM; Walsh, 2003) and Metaphor Theory (MT; Lakoff and Johnson, 1999). These theories lead to different predictions about exactly *how* space and time are related in our brains and minds.

According to ATOM, space, time and other prothetic domains^1^ such as numerosity and brightness are represented in the brain and mind by a common analog magnitude system. Support for ATOM comes from behavioral experiments showing cross-dimensional priming or interference between different prothetic domains (Henik and Tzelgov, 1982; Dormal and Pesenti, 2007; Bueti and Walsh, 2009), and from neuroimaging studies showing that magnitude processing in various domains activates overlapping areas in the parietal lobe (Fias et al., 2003; Pinel et al., 2004; Dormal and Pesenti, 2009; cf., Gijssels et al., 2013).

Implicit in ATOM is an assumption that these “ATOMic” domains are symmetrically interrelated. Accordingly, Walsh (2003) frames predictions in symmetrical terms, positing “overlapping brain regions” and “cross-domain, within-magnitude priming,” without specifying any *directionality* to the priming or interference effects. If space and time are both represented by the same general-purpose analog magnitude metric, there is no *a priori* reason to posit that representations in one domain should depend asymmetrically on representations in the other.

By contrast, according to MT (e.g., Lakoff and Johnson, 1999), representations of time and number depend asymmetrically on representations of space. The claim that some domains tend to be asymmetrically dependent on others in our mental representations, which is at the core of MT, was originally supported by patterns in metaphorical language. In English, it is nearly impossible to talk about domains like time without using words whose primary meaning is spatial [denotatively, developmentally, or historically (Clark, 1973)]. Vacations can be *long* or *short*, meetings can be *moved forward* or *pushed back*, deadlines can loom *ahead* or lie *behind* us. Although it is also possible to use temporal words to talk about space, time-to-space mappings in language are far less common than space-to-time mappings (Lakoff and Johnson, 1999). This asymmetry in language has been echoed by behavioral findings in psycholinguistics (Boroditsky, 2000), cognitive development, (Casasanto et al., 2010), and psychophysics (Casasanto and Boroditsky, 2008; Bottini and Casasanto, 2010; Merritt et al., 2010), and also by asymmetric activation of the hypothetical “ATOM area” in the brain, the Inferior Parietal Cortex, during encoding of spatial vs. temporal information (Gijssels et al., 2013).

In the first set of psychophysical studies that was designed to test for a space-time asymmetry, participants viewed lines of various spatial lengths that appeared on a screen for varying durations (Casasanto and Boroditsky, 2008). They were asked to estimate either the duration or the spatial length of each line by clicking the mouse to indicate the starting and ending points of each spatial or temporal interval. Spatial and temporal extents were fully crossed in the stimuli. Results showed that participants were unable to ignore irrelevant spatial information when making judgments about duration, but not the converse. For stimuli of the same average duration, lines that extended a shorter distance in space were judged to take a shorter time, and lines that extended a longer distance were judged to take a longer time. By contrast, for stimuli of the same average spatial length, spatial estimation was not affected by the line's duration. The cross-domain asymmetry that was predicted on the basis of patterns in language was found in non-linguistic psychophysical judgments. Five follow-up experiments varied the attentional, mnemonic, and perceptual demands of the stimuli, and all six experiments supported the same conclusion: mental representations of time depend on representations of space, more than vice versa.

This robust space-time asymmetry supports MT, but presents a challenge to ATOM. If spatial and temporal extent are both represented by a general-purpose magnitude metric, then why should representations of time depend on representations of space more than the other way around—in adults and children, and in language and thought? Proponents of ATOM have acknowledged that cross-domain asymmetries are problematic for the theory, and have argued that perhaps observed asymmetries could be attributed to task-related factors, such as differences in the discriminability of the stimuli across dimensions. According to Bueti and Walsh (2009) “whether these findings are evidence of constant asymmetries or are task dependent remains to be established” (p. 1833; see also Lourenco and Longo, 2011).

We agree that some of the cross-domains asymmetries that have been reported may be artifacts of imbalances built into the stimuli or the tasks, especially in studies that were not designed to test for cross-domain asymmetries (e.g., Dormal and Pesenti, 2007). But several studies have been designed expressly for this purpose, using Garner-like interference tasks (Garner, 1976) in which participants judged either the spatial or the temporal dimension of each stimulus, while attempting to ignore the irrelevant dimension (e.g., Casasanto and Boroditsky, 2008; Casasanto et al., 2010; Merritt et al., 2010; Gijssels et al., 2013). The discriminability of stimuli and the complexity of the responses in each domain were controlled. Participants' accuracy within each domain was matched to ensure that cross-domain asymmetries were not the result of judgments in one domain being “messy” (and therefore more susceptible to cross-dimensional interference) and judgments in the other domain being relatively “clean” (and therefore resistant to cross-dimensional interference). In short, these studies were designed to eliminate task-related asymmetries between domains, and numerous theory-irrelevant explanations for the observed space-time asymmetries were ruled out through a series of experiments spanning several years and several papers.

Still, we agree with Bueti and Walsh (2009) that cross-domain asymmetries should only be considered challenges to ATOM to the extent that theory-irrelevant task-related causes of the asymmetry can be ruled out. To this end, in the present study we seek to address a potential weakness of the task used by Casasanto et al. (2010) to test for a space-time asymmetry in children. In the original study children watched movies of two snails traveling along parallel paths for different distances and durations. At the end of each movie the children were asked to judge which of the two snails moved for a longer time or for a longer distance. Spatial and temporal distances were fully crossed in the stimuli. Results showed that temporal judgments were influenced by the spatial information more than vice versa, consistent with the results of the analogous psychophysical tasks in adults.

Casasanto et al. (2010) noted that one aspect of their stimuli merited further consideration. On the critical trials testing for cross-dimensional interference, children received exactly the same spatial and temporal information before answering either the spatial or the temporal questions (i.e., they saw the same movies of racing snails). This task was a digital adaptation of a task Piaget (1927/1969) conducted using mechanical snails racing across a physical surface. The authors made an effort to preserve important regularities of the physical world in their digital facsimile. As a result, the snails remained on the screen in their final “resting” positions at the end of the race (as wind-up snails would if they were racing across a real tabletop). Therefore, children had a persistent, visible spatial cue to refer to when judging the relative distance of the snails, but no persistent, visible temporal cue when judging their relative duration. This fact about the stimuli reflects a simple fact of the physical world: unlike spatial relationships, which can often be inspected and reinspected, temporal relationships are by their nature neither persistent nor visible.

Could this feature of the stimuli be responsible for the observed cross-dimensional asymmetry? This is unlikely to be the case, since none of the otherwise analogous psychophysical experiments in adults had this potential problem. But we cannot rule out the possibility that the space-time asymmetry reported by Casasanto et al. (2010) was produced (or enhanced) by this imbalance in the perceptual availability of spatial and temporal information. Here we conducted a stronger test of the hypothesized space-time asymmetry in children, controlling spatial and temporal aspects of the stimuli even more stringently than they are “controlled” in the natural world. In this version of the experiment, the two snails disappeared at the end of the race, as soon as they had both stopped moving. Therefore, no visible record of the distance the snails had traveled remained during the question period, equating the perceptual availability of spatial and temporal information. If the persistence of spatial (but not temporal) information on the screen at the end of the snail races was responsible for the observed space-time asymmetry in the earlier study, the asymmetry should be attenuated or extinguished in the present version of the experiment. Alternatively, if the observed space-time asymmetry found previously reflected the way space and time tend to be related in children's mental representations of motion events, then the asymmetry should persist.

## Method

### Participants

Fifty-six children (4–10 years old) participated to the experiment. Half of the children were native Dutch speakers living in the Netherlands, and the other half were native Portuguese speakers living in Brazil. Dutch children ranged in age from 4- to 9-year-old (Mean = 6.6, *SD* = 1.8), Brazilian children from 6 - to 10-year-old (Mean = 7.7, *SD* = 1.4).

The study has been conducted under the strict respect of the World Medical Association's Declaration of Helsinki. Children participated in the experiments after receiving the express written consent from their parents.

### Materials and procedure

The materials and procedure were identical to those used by Casasanto et al. (2010) with the following exceptions. First, in the original experiment the stimuli remained on the screen while children responded to the experimenter's questions; in this version of the experiment the stimuli were removed from the screen during the question period. Second, materials were translated into Dutch for children tested in The Netherlands, and into Portuguese for the children tested in Brazil.

The experiment had a 2 × 2 × 2 × 2 design: Target Dimension (Space, Time), and Dimensional Interference (Cross-Dimensional Interference, No Cross- Dimensional Interference) were within-subjects factors, and Age (Younger, Older) and Language (Dutch, Portuguese) were between-subjects factors. In the cross-dimensional interference tasks children were required to make either a temporal or spatial judgment in presence of competing information from the other dimension. For the non-interference tasks, children judged distance or duration without any competing information from the other dimension.

Each participant performed three tasks: Racing Snails (Distance–Time interference task), Jumping Snails (to test children's ability to judge duration independent of spatial interference), and Static Lines (to test children's ability to judge distance independent of temporal interference). Each task is described below.

Stimuli were presented on a Macintosh laptop (resolution = 1024 × 768 pixels) and were followed by written questions (displayed for the experimenter's benefit). The first question of each trial was intended to focus children's attention on the stimulus event and to allow the experimenter to evaluate whether the child was paying attention. The second question, which asked children to judge either relative distance or relative duration, was of critical interest.

The experiments were conducted in the children native language by a native Dutch speaker for children tested in the Netherlands and a native Portuguese speaker for children tested in Brazil. Children were tested individually at their schools, in a private room away from other children. Each child completed a total of 18 trials (12 cross-dimensional interference trials and six no-interference control trials). Testing lasted about 10–15 min.

#### Racing snails (space-time interference task)

Two snails, one above the other, began at the left edge of the screen and “raced” rightward along parallel tracks. One snail was blue and the other red, so that they would be visually discriminable and easy for the child to name (e.g., “the blue one”). The assignment of colors to the top and bottom snails was counterbalanced across participants.

There were three types of movies, placing the snails in different space–time relationships relative to one another. The two snails traveled: (a) Different distance, different time, (b) Different distance, same time, or (c) Same distance, different time. Distances traveled were either 400 or 600 pixels, and durations of travel were either 4 or 6 s. There were two variants of each movie type, in which either the top or the bottom snail traveled longer in space or time. This control was implemented in case participants who had an overall preference to choose the snail on the top or the bottom. This resulted in six movies that could be viewed serially without repetition.

Each participant saw all six of the Racing Snails movies twice, once in each of two blocks: a Time Question block and a Space Question block. The order of movies within each block was randomized.

At the beginning of each question block the experimenter encouraged the child to pay attention either to the distance traveled by the snails or to the duration of the snails' movement. In the space question block, after each movie, the participant was asked two questions: 1 “*Did the two snails stop at the same place?*” 2. “*Did one of the snails go farther?*” and if the child responded affirmatively without specifying which snails had gone farther, the experimenter asked: “*Which one of the two?*” In the time question block children saw the same 6 movies, but were asked about the temporal aspect of the snails' movement. After each movie the experiment asked: 1 “*Did the two snails stop at the same time?*” 2. “*Did one of the snails move for a longer time?*” and if necessary, “*Which one?*”

In the previous version of this experiment (Casasanto et al., 2010) all stimuli remained in their final resting positions until after the child responded. In this version of the experiment the two snails disappeared when both had stopped moving.

#### Static lines (distance estimation control task)

The static lines task was used to test children's ability to make distance judgments without any competing temporal information. Children judged three pairs of static lines presented one pair at a time, one above the other. One line was red and the other blue, with the colors of the top and bottom lines counterbalanced across participants. The lines were either 400 or 600 pixels in length and came in three combinations: (a) top line longer, (b) bottom line longer, or (c) both lines the same length (600 pixels). The experimenter asked: 1. “*Are the lines the same length?*” 2. “*Is one of the lines longer?*” and if necessary, “*Which one is longer?*”

#### Bouncing snails (duration estimation control task)

The bouncing snails task tested children's ability to make duration judgments without any competing distance information. Children judged three movies of the red and blue snails bouncing up and down in place, one above the other. The colors of the top and bottom snails were counterbalanced across participants. Each of the snails bounced for either 4 or 6 s, in one of three combinations: (a) top snail bounced longer, (b) bottom snail bounced longer, or (c) both snails bounced for the same duration (6 s). Although the snails traveled a small distance up and down while bouncing, there was no lateral motion and no net displacement. The experimenter asked: 1. “*Did the two snails stop at the same time?*” 2. “*Did one of the snails move for a longer time?*” and if necessary, “*Which one?*”

## Results

We analyzed the proportion of correct responses from each of the two groups of participants (Dutch, Brazilian) with a multiple linear regression with Target Dimension (Space, Time), Interference (With Interference, Without Interference) and the interaction of these two factors as predictors.

**Figure 1:**
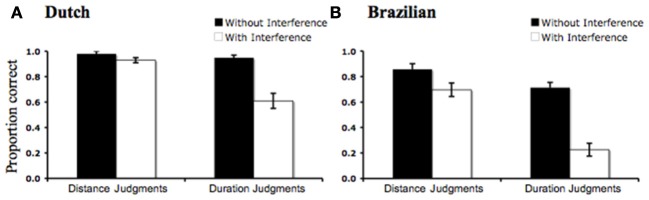
**Effect of cross-dimensional interference on spatial and temporal judgments for Dutch (A) and Brazilian (B) children.** In both cases the effect of distance interference on duration judgments was greater than the effect of duration interference on distance judgments. Error bars show s.e.m.

In both groups there was a main effect of Interference [Dutch: *F*_(1)_ = 26.99, *p* = 0.001, *r*^2^ = 0.16; Brazilian: *F*_(1)_ = 34.13, *p* = 0.001, *r*^2^ = 0.19], indicating better performance during the no-interference tasks (Jumping Snails and Static Lines) than during the cross-dimensional interference task (Racing Snails). Additionally, there was a main effect of Target Dimension [Dutch: *F*_(1)_ = 22.19, *p* = 0.001, *r*^2^ = 0.13; Brazilian: *F*_(1)_ = 30.48, *p* = 0.001, *r*^2^ = 0.17], indicating better performance during Space trials compared to Time trials, overall. Crucially, there was also a highly significant interaction of Interference and Target Dimension [Dutch: *F*_(1)_ = 16.53, *p* = 0.001, *r*^2^ = 0.09; Brazilian: *F*_(1)_ = 8.69, *p* = 0.003, *r*^2^ = 0.05], indicating that the effect of cross-dimensional interference was much greater for duration judgments than for distance judgments (Figure 1).

To analyze the effect of age on the magnitude of the cross-dimensional asymmetry, we divided both groups of participants in younger (≤7 years old) and older (>7 years old) children and added to the model the factor Age (Younger, Older) letting it interact with Target Dimension and Interference. Crucially, the three way interaction of Target Dimension, Interference and Age did not approach significance for either group [Dutch: *F*_(1)_ = 0.42, n.s., *r*^2^ = 0.001; Brazilian: *F*_(1)_ = 1.91, n.s., *r*^2^ = 0.01], indicating that the strength of the space-time asymmetry did not change significantly with age (consistent with Casasanto et al.'s (2010) results).

**Figure 2:**
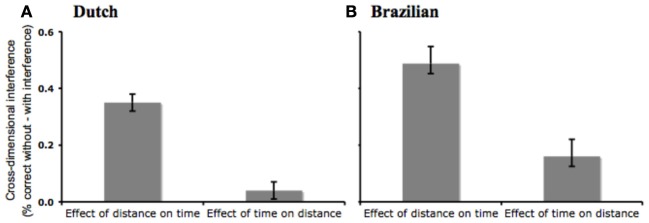
**Comparison of cross-dimensional interference effects for Dutch (A) and Brazilian (B) children.** Error bars indicate s.e.m.

Next, we tested whether the magnitude of the space-time asymmetry varied across language groups. We run a multiple linear regression with Interference (With Interference, Without Interference), Target Dimension (Space, Time), Language (Dutch, Portuguese) and their interactions as predictors of the proportion of correct responses. There was a main effect of Language [*F*_(1)_ = 53.19, *p* = 0.001, *r*^2^ = 0.18], indicating that Dutch children were over-all more accurate than Brazilian ones. Nevertheless, the critical three-way interaction of Target Dimension, Interference and Language did not approach significance [*F*_(1)_ = 0.30, n.s., *r*^2^ = 0.001], indicating that the magnitude of the space-time asymmetry did not vary across language groups (Figure 2).

Did the observed space-time asymmetry arise because space judgments were easier than time judgments (as indicated by the main effect of Target Dimension)? The fact that we found a highly significant interaction of Interference by Target Domain in each language group of participants argues against this possibility. Children were not simply bad at judging time and good at judging space: they were particularl*y* bad at judging time *in the presence of spatial interference* (much more than vice-versa).

For instance, a comparison of the Dutch children's accuracy in the Static Lines vs. Bouncing Snails tasks suggests that they were about equally good at judging space *per se* and time *per se* [*t*_(27)_ = 1.44, *p* = 0.16]; yet, they showed a highly significant cross-dimensional asymmetry. Could the critical interaction of Interference by Target Domain that demonstrates this asymmetry be created by a ceiling effect in the non-interference conditions? This is unlikely: Brazilian children's performance was not at ceiling (i.e., performance was significantly lower than 100% accurate) in the Bouncing Snails task [*t*_(27)_ = 4.49, *p* = 0.001, one-tailed] or the Static Lines task [*t*_(27)_ = 3.27, *p* = 0.001, one-tailed], yet they show the predicted cross-dimensional asymmetry.

Brazilian children were significantly better at judging space *per se* (Static Lines) than time *per se* [Bouncing Snails; *t*_(27)_ = 2.57, *p* = 0.02]. Could the difference in performance between spatial and temporal judgments be responsible for the critical space-time asymmetry observed in the Racing Snails task? To provide further evidence against this possibility, we conducted an analysis in which we equated performance on the spatial and temporal control tasks, including only those Brazilian children who performed perfectly on both the Jumping Snails and Static Lines tasks (*n* = 12). In this subsample, spatial judgments were still more accurate than temporal judgments in the Racing Snails task [*F*_(1)_ = 19.76, *p* = 0.001, *r*^2^ = 0.13]. Even when children were matched on their ability to judge relative distance and relative duration, *per se*, their judgments under cross-dimensional interference conditions revealed the predicted space-time asymmetry.

## Discussion

When asked to judge spatial or temporal extent, children were much better at judging distance in the presence of temporal interference than judging duration in the presence of spatial interference. This asymmetric pattern of cross-dimensional interference was found even in children who were 100% accurate when asked to judge spatial and temporal extent, *per se*, in the absence of competing information from the irrelevant dimension (i.e., in the control conditions). This finding replicates the results of Casasanto et al. (2010), and rules out the skeptical possibility that the cross-dimensional asymmetry reported in this earlier study was due to the fact that spatial information remained visible while children were responding but temporal information did not. Furthermore, the present results extend the earlier findings in Greek children to Dutch and Brazilian children, providing evidence of the generality of the space-time asymmetry in children's minds.

### Is the space-time asymmetry due to cross-domain differences in the perceptual discriminability of spatial and temporal stimuli?

Did the space-time asymmetry, found here and in previous experiments, arise because space was more discriminable than time in the stimuli? There is evidence that this is not the case. Discriminability refers to the psychological difference between stimulus values along a dimension (Melara and Mounts, 1993; Algom et al., 1996). Two dimensions are equally discriminable when the values along one dimension are equally different psychologically compared to the values along the other dimension (Melara and Mounts, 1993). In principle, differences in discriminability between space and time could complicate the interpretation of cross-dimensional interference effects, since differences in discriminability can give rise to asymmetric patterns of interference, with the more discriminable domain interfering with the less discriminable one more than vice versa (Melara and Mounts, 1993; Algom et al., 1996; Pansky and Algom, 1999).

Differences in discriminability correspond to differences in the accuracy, precision, speed or variability of judgments across domains (Melara and Mounts, 1993; Algom et al., 1996; Pansky and Algom, 1999; Santiago et al., 2011). Importantly, in previous studies supporting the space-time asymmetry (Casasanto and Boroditsky, 2008; Casasanto et al., 2010; Merritt et al., 2010), within-domain performance was *equivalent* across space and time. That is, judgments of duration were just as precise and accurate as judgments of spatial length. Similarly, in the current experiment, Dutch children were equally accurate in judging time *per se* and space *per se*, yet they showed the predicted space-time asymmetry. Brazilian children, who showed better accuracy in spatial judgments overall, continued showing the predicted asymmetry when their performance on temporal and spatial judgments was matched.

The question of whether the space-time asymmetry found in humans depends on asymmetries in the perceptual discriminability of spatial and temporal stimuli was addressed decisively in an experiment by Bottini and Casasanto (2010). In this experiment, the possibility of differences in the perceptual discriminability of spatial vs. temporal stimuli was completely eliminated. Dutch-speaking participants saw 7- letter nouns that named concrete objects of various spatial lengths (*tr.* pencil, bench, clothesline) and estimated how much time they remained on the screen. A different group of participants saw nouns naming temporal events of various durations (*tr.* blink, party, season) and estimated the words' spatial length. The implicit length encoded in object nouns modulated time estimates, but the implicit duration encoded in event nouns did not affect estimates of spatial length. Nouns that named short objects were judged to remain on the screen for a shorter time, and nouns that named longer objects to remain for a longer time. By contrast, variations in the duration of the event nouns' referents had no effect on judgments of the words' spatial length on the screen. Since in this study there was no perceptible variation of the interfering domain (space and time only varied implicitly, according to the length of the words' referents), the asymmetric pattern of cross-dimensional interference cannot be attributed to differences in the perceptual discriminability of space and time in the stimuli.

Further evidence that the space-time asymmetry is due to the way these dimensions tend to be represented in the human mind, and not to task-related differences in perceptual discriminability across domains, comes from a study with non-human primates. Merritt et al. (2010) had both humans and rhesus macaques (Macaca mulatta) perform Garner-like space-time interference tasks. Discrimination sensitivities were similar for space and time in both monkeys and humans (as measured by the comparison of Weber fractions), but humans showed the expected space-time asymmetry whereas monkeys did not. Space-time interference was symmetric in monkeys but asymmetric in humans even though spatial and temporal judgments were matched for discriminability in both groups, demonstrating that that the cross-dimensional asymmetry can appear (or not appear) independently of the relative discriminability of space and time.

More fundamentally, Merritt et al.'s (2010) comparison of monkeys and humans definitively rules our any possible explanation for the humans' space-time asymmetry in terms of an imbalance between space and time inherent in the stimuli. Given the same stimuli and the same task, monkeys showed symmetric space-time interference (with the pattern trending, in fact, toward the *opposite* of the expected asymmetry), whereas humans showed the expected space-time asymmetry. In the experiments reviewed above, the locus of the space-time asymmetry is not in the stimuli or the tasks, but rather in the human observers' minds.

To summarize this point, although differences in discriminability across perceptual domains can cause asymmetric patterns of cross-domain interference (Melara and Mounts, 1993; Algom et al., 1996; Pansky and Algom, 1999, 2002) this was not the cause of the space-time asymmetry observed across several studies that were expressly designed to test for this asymmetry. The asymmetric dependence of time on space in people's judgments is not an artifact of perceptual asymmetries built into the stimuli or the tasks. Rather, this performance asymmetry reflects the way space and time tend to be represented in the human mind.

### Do *Tau* and *Kappa* effects constitute evidence against metaphor theory?

The *Tau* and *Kappa* effects have been repeatedly observed in several psychophysical experiments (Benussi, 1913; Cohen et al., 1954; Price-Williams, 1954; Jones and Huang, 1982; Sarrazin et al., 2004). In the classic setup participants are asked to compare two spatiotemporal intervals delimited by three light bulbs that are rapidly flashed in succession. When participants are asked to compare the duration of the two intervals, their judgments are modulated by the spatial distance between the flashes: greater distances correspond to greater duration judgments (Kappa effect). Similarly, distance judgments also increase as a function of the duration between successive flashes (Tau effect). Together, the Tau and Kappa effects are sometimes interpreted as inconsistent with the space-time asymmetry predicted by MT (Lourenco and Longo, 2011).

At first glance, the existence of both Tau and Kappa effects may seem at odds with a space-time asymmetry, but these findings can be easily reconciled. First, MT can accommodate Tau-like effects since it predicts an *asymmetrical* (hence bidirectional) relationship between space and time, not a *unidirectional* one. The prediction of asymmetry—not unidirectionality—has been explicit since MT's inception (Lakoff and Johnson, 1980), and is motivated by the asymmetry (not unidirectionality) of source and target domains in linguistic metaphors. It is very common and nearly obligatory in English to use spatial expressions for time, it is also possible (albeit far less common) to use temporal expressions for space (e.g., “we're just a few blocks from the station” can mean “it will take us a short time to reach the station” Casasanto and Boroditsky, 2008). MT assumes *bidirectional* transfer between source and target domains, which is more frequent, productive, and automatic in one direction than the other. Effects of time on space are not at odds with MT, so long as (under adequately controlled conditions) a *greater* or *more frequent* effect of space on time is also found.

Second, as suggested by Casasanto and Boroditsky (2008), the Tau and Kappa effects are no challenge to the space-time asymmetry whatsoever if, as the leading account of these effects suggests, they do not show effects of space on time or of time on space, *per se*. Although a complete explanation of the Tau and Kappa effects is still missing, the most successful account of this effect is the “Imputed Velocity Hypothesis” (Jones and Huang, 1982). On this view Tau and Kappa effects arise because “subjects impute uniform motion to discontinuous displays” (Jones and Huang, 1982, p. 183). That is, the succession of discrete flashes is computed as the continuous movement of one flash, to which a constant velocity is intuitively attributed. The attribution of a constant velocity to the apparently moving stimulus creates the illusion that the stimulus is moving for a farther distance when the duration between the flashes is extended, or that the motion takes a longer time when the distance between flashes is extended. The Imputed Velocity Hypothesis is supported by several experiments showing the effect of acceleration patterns on the magnitude of Kappa and Tau effects, both in vision and audition (Jones and Huang, 1982; Henry and McAuley, 2009; Henry et al., 2009).

In conclusion, Tau and Kappa effects seem to be effects of imputed velocity on judgments of both time and space, rather than mutual effects between temporal and spatial aspects of the events, and are therefore irrelevant to the question of whether space and time have symmetric or asymmetric effects on one another (Casasanto and Boroditsky, 2008).

### Can time ever influence space in our minds?

There is no question that time can influence spatial thoughts and behaviors under some circumstances, just as time sometimes serves as a source domain for space in metaphorical language (see §5.2). But as we have argued above, the simple fact of a bidirectional relationship between space and time in language, thought, and behavior is no challenge to the claim that time is asymmetrically dependent on space in our minds, which is supported by a variety of evidence suggesting that the use of space to represent time is more frequent, flexible, and automatic than the use of time to represent space.

It would be truly extraordinary if the relationship between space and time were unidirectional in light of the pervasive bidirectionality of neural systems (e.g., Lamme and Roelfsema, 2000), and of natural systems more broadly. Consider even a very simple natural system like a chemical reaction in which two reactants are very strongly driven to form a product (indicated in chemical formalism by a large forward arrow pointing from reactants to product). Even in such cases, there is *some* reformation of the reactants (indicated by a small backwards arrow from the product to the reactants).

When should we expect time to influence space in our minds? To answer this question, it is useful to consider why the observed representational asymmetry exists. Metaphor Theorists are careful to point out that space and time are *equally basic* in our experience of the world, and that extent in space and in time are correlated with one another—correlation being an inherently symmetrical relationship (Lakoff and Johnson, 1980; Casasanto et al., 2010). Yet, despite being symmetrically related in the word, space and time are asymmetrically represented in our brains (Gijssels et al., 2013) and behaviors (e.g., Casasanto and Boroditsky, 2008). Perhaps we tend to rely asymmetrically on the dimension that is easier to perceive, remember, or reconstruct from physical evidence. That is, we may use space heuristically as an index of time because, in many cases, the spatial dimension of an event is more accessible than the temporal. (“Accessibility” could the mean greater familiarity, perceptual availability, imageability, memorability, etc.). Alternatively, if contexts exist in which space is not more accessible than time we might not rely on space as an index of time—at least not as much as we ordinarily do.

### Are space and time symmetrically related in infants' minds?

Evidence for a strong space-time asymmetry in kindergarteners does not rule out the possibility that space and time could be symmetrically related in infants' minds, and that the relationship between the domains becomes asymmetrical over the course of cognitive development (Casasanto et al., 2010). Lourenco and Longo (2010) suggested that space and time (and number) may be symmetrically interrelated in the minds of 9-month-old infants. In their experiment, infants were trained to associate specific colors and patterns with high or low magnitudes within a single dimension (e.g., black objects with stripes were large in size; white objects with spots were small in size). After the training on one dimension (e.g., size), participants saw similar objects that varied in magnitude along another dimension (e.g., duration of presentation). The mapping from color/pattern to magnitude was either preserved (congruent condition) or reversed (incongruent condition) between the training domain and the test domain. Results showed that infants spent more time looking at test objects with incongruent mappings compared to congruent ones, suggesting that they were generalizing the mapping across domains (e.g., from space to time). Crucially for the present discussion, the process of generalization between space and time was symmetric: there was no difference in the size of the congruity effect when infants were generalizing from time to space vs. from space to time. According to Lourenco and Longo (2010, 2011), this symmetric cross-domain transfer challenges MT and supports ATOM.

Yet, it may be premature to interpret these data as evidence for a common analog magnitude metric underlying representations of duration, size, and number. In order to interpret these data as support for ATOM, it would be necessary to show that Lourenco and Longo's task was a valid test of the relationships between prothetic domains in infants' minds (i.e., a valid test of their symmetry or asymmetry). At present, the study is difficult to interpret because it is missing both a manipulation check and a control condition.

An appropriate manipulation check would demonstrate that this task *can* reveal a significant asymmetry in transfer between domains where such an asymmetry exists in participants' mental representations. In the absence of a manipulation check, it is hard to interpret these data as evidence against a cross-dimensional asymmetry because the authors' tests for asymmetries in the amount of transfer between domains (e.g., from space to time vs. from time to space) produced null results.

ATOM posits a shared representational substrate for prothetic domains, only. Therefore, an appropriate control condition would show that the pattern of transfer found for prothetic domains *would not be found* in tests of transfer between non-prothetic domains (e.g., transfer between two metathetic domains, or between a prothetic and metathetic domain). For example, in principle Lourenco and Longo's paradigm could show that infants transfer greater size not only to objects with greater duration (i.e., transfer between two prothetic domains) but also to objects that produce *lower pitches* (i.e., transfer between a prothetic and a metathetic domain). This result would be expected in light of evidence from another preferential looking study showing that 4-month-olds intuit a relationship between larger size and lower pitch (Dolscheid et al., 2012). High and low pitches do not differ from one another in *magnitude* (when their loudness and duration are equated); therefore the mapping from size to pitch cannot be based on activation of a common magnitude metric. If a size-pitch version of Lourenco and Longo's task showed a similar result to Dolscheid et al.'s, this would indicate that their size-time-number data cannot be interpreted as evidence of an ATOMic generalized magnitude system. Rather, their results could indicate a broader capacity for transferring associations across domains.

It remains an open question whether space and time are linked symmetrically or asymmetrically in infants' minds; that is, whether space-time representations start out ATOMic and only later become metaphoric. Garner-like interference paradigms may provide the best tool to investigate whether the space-time relationship is symmetric or asymmetric (Garner, 1976; Casasanto and Boroditsky, 2008; Casasanto et al., 2010; Merritt et al., 2010; Gijssels et al., 2013). Adapting Garner-like tasks for experiments with infants remains a challenge for future research.

## Conclusions

Space and time are related asymmetrically in children's minds. Kindergarten and elementary school–aged children can ignore irrelevant temporal information when making judgments about space, but they have difficulty ignoring spatial information when making judgments about time. This asymmetric relationship, which was predicted on the basis of patterns in metaphorical language, does not depend on a task-related difference in the perceptual availability of spatial and temporal information in the stimuli. Results replicate those of Casasanto et al.'s (2010) study in Greek children, strengthening the finding by removing a possible confound in the earlier study, and extending it to children who speak Dutch and Brazilian Portuguese. The observed space-time asymmetry supports MT, which suggests that people often use space as a representational scaffold for time, but it challenges ATOM, which accords equal status to representations of space, time, and other prothetic dimensions.

In principle, it is possible that ATOM could be modified to accommodate a representational asymmetry between space and time. For example, in his initial ATOM proposal, Walsh (2003) emphasized that temporal, spatial, and numerical magnitudes should all be computed by a “common metric,” but he also mentioned in passing that “the [general] magnitude system appears to have a spatial basis” (p. 486). If so—if the “common metric” that ATOM proposes is space, and numerical and temporal magnitudes are represented via spatial magnitude—then perhaps ATOM *should* predict a space-time asymmetry. But if the common metric in ATOM is space, then ATOM's most basic claim becomes: there is a spatial basis for time and number—a claim that is indistinguishable from MT's.

### Conflict of interest statement

The authors declare that the research was conducted in the absence of any commercial or financial relationships that could be construed as a potential conflict of interest.

## Footnotes

^1^Prothetic domains are domains in which people can experience quantitative variation (e.g., more or less size, duration, brightness, etc.), whereas only qualitative variation is possible within metathetic domains (e.g., hue, pitch, timbre; Stevens, 1957).
